# Epidemic spread of adenovirus type 4-associated acute respiratory disease between U.S. Army installations.

**DOI:** 10.3201/eid0604.000419

**Published:** 2000

**Authors:** K M McNeill, F Ridgely Benton, S C Monteith, M A Tuchscherer, J C Gaydos

**Affiliations:** Catawba Health District, South Carolina Department of Health and Environmental Control, Lancaster, South Carolina 29721, USA. mcneilkm@lncstr60.dhec.state.us

## Abstract

A large outbreak of adenovirus type 4-associated acute respiratory disease (ARD) occurred at Fort Jackson, South Carolina, in 1997. A laboratory-based ARD surveillance program was initiated at Fort Gordon, Georgia, where advanced individual training was heavily populated with Fort Jackson soldiers. Adenovirus type 4 was isolated from 50% of 147 trainees hospitalized with ARD. Most (88%) introduced cases were in trainees from Fort Jackson.


### Epidemic Spread of Adenovirus Type 4-Associated Acute Respiratory Disease between U.S. Army Installations

**K. Mills McNeill,* F. Ridgely Benton,† Susan C. Monteith,† Margaret A. Tuchscherer,† and Joel C. Gaydos‡**
*Catawba Health District, South Carolina Department of Health and Environmental Control, Lancaster, South Carolina, USA; † Dwight David Eisenhower Army Medical Center, Fort Gordon, Georgia, USA; ‡U.S. Department of Defense Global Emerging Infections Surveillance and Response System, Silver Spring, Maryland, USA

A large outbreak of adenovirus type 4-associated acute respiratory disease (ARD) occurred at Fort Jackson, South Carolina, in 1997. A laboratory-based ARD surveillance program was initiated at Fort Gordon, Georgia, where advanced individual training was heavily populated with Fort Jackson soldiers. Adenovirus type 4 was isolated from 50% of 147 trainees hospitalized with ARD. Most (88%) introduced cases were in trainees from Fort Jackson.

A large outbreak of adenovirus type 4-associated acute respiratory disease (ARD) occurred at Fort Jackson, South Carolina, the U.S. Army's largest basic training center (1) from May through December 1997. During the latter half of 1997, ARD hospitalizations were reported in basic trainee populations from all army training centers. However, the highest rates (≥1% of all trainees per week) were at Fort Jackson and at Fort Leonard Wood, Missouri (2).

After completing an 8-week basic training, soldiers generally enter the second phase of military specialty training, advanced individual training, which is offered at various U.S. Army posts. Fort Gordon, Georgia, receives soldiers directly from Fort Jackson (approximately 150 km away) and other basic training centers. From August to December 1997, the average advanced training population at Fort Gordon was 3,600 soldiers; 80% came from Fort Jackson, approximately 10% from Fort Leonard Wood, and 10% from other sites. Most soldiers proceed directly to advanced training upon graduation from basic training at Fort Jackson. Concern that adenovirus-associated ARD might spread to advanced training students at Fort Gordon led to an intensive, laboratory-based ARD surveillance program at this site on April 1, 1997.

### The Study

Female and male soldiers in advanced training live in barracks at Fort Gordon and receive medical care at the army hospital. Soldiers report to outpatient clinics during the day or to the emergency department during evenings and weekends. Local medical policy requires hospitalization of advanced training soldiers with ARD symptoms (one or more signs or symptoms of acute respiratory infection and oral temperature of ≥38.06°C). Approximately 80% of advanced training soldiers admitted to the Fort Gordon hospital who met the case definition were enrolled in the surveillance program.

A questionnaire was used to record basic training site, date of reporting to Fort Gordon, and date of onset of symptoms for each trainee. A pharyngeal swab was taken from each trainee, placed directly into virus transport medium (Viromed Laboratories, Inc., Minneapolis, MN), and immediately transported to the laboratory at the Dwight David Eisenhower Army Medical Center, Fort Gordon. All procedures performed on human subjects met ethical standards

Address for correspondence: K. Mills McNeill, Catawba Health District, South Carolina Department of Health and Environmental Control, P.O. Box 817, Lancaster, SC 29721, USA; fax: 803-286-5418; e-mail: mcneilkm@lncstr60.dhec.state.us.

established by the Institutional Review Committee of the medical center.

Viruses were isolated and identified in the Virus Isolation Laboratory, Dwight David Eisenhower Army Medical Center. Adenoviruses were isolated on human lung carcinoma (A-549) cells. Serotypes were determined by virus neutralization (3) with type-specific antisera (Centers for Disease Control and Prevention, Atlanta, GA) by the Reed-Muench method for calculation of the 50% lethal dose titer (4). Cultures were also examined by standard virus isolation and identification for influenza A and B; parainfluenza 1, 2, and 3; herpes simplex; and enteroviruses. In addition to human lung carcinoma, primary rhesus monkey kidney and human foreskin cell lines were used to screen for viral agents. A pharyngeal swab was obtained from most study participants for isolation of *Streptococcus pyogenes*, Group A, by using standard methods.

The incubation period for adenovirus ARD is generally ≤10 days (5,6). Patients who had been at Fort Gordon for ≤10 days before the onset of ARD symptoms were classified as having been infected elsewhere (introduced cases). Patients who had been at Fort Gordon for >10 days before onset were classified as having acquired adenovirus infection at Fort Gordon (secondary cases).

From April 17 to May 14, 1997, a survey was undertaken to determine if adenoviruses were circulating in trainees who did not exhibit classic ARD. Throat swabs for virus isolation were performed on 180 randomly selected Fort Gordon advanced training soldiers who reported to clinic for treatment of minor illnesses, including afebrile upper respiratory infections.

The first isolate of adenovirus type 4 was from a specimen collected on August 19, 1997, from an advanced training soldier whose illness met the case definition of ARD. None of the specimens obtained from advanced training soldiers without ARD yielded adenovirus. From August 1, 1997, through December 31, 1997, 147 advanced training cases from four basic training sites that met the ARD case definition were studied; 73 (49.7%) patients had positive test results for adenovirus type 4. Each began basic training after adenovirus vaccination had ceased. Of the 73, 7 (9.6%) completed basic training at Fort Leonard Wood; 6 (8.2%) trained at Fort Sill, Oklahoma; 3 (4.1%) trained at Fort Knox, Kentucky; and 57 (78.1%) trained at Fort Jackson. The 57 adenovirus type 4-positive soldiers included 47 (82.5%) men and 10 (17.5%) women with a median age of 19 years (range 17 to 33 years). This gender distribution closely mirrored that of the total advanced training population at Fort Gordon (approximately 80% male and 20% female).

Of 119 cases in soldiers who had performed basic training at Fort Jackson, 57 (47.9%) were adenovirus type-4 positive; of these cases, 22 (38.6%) were introduced and the remaining 35 (61.4%) were attributed to local transmission at Fort Gordon (Figure 1). Thirty-three other ARD

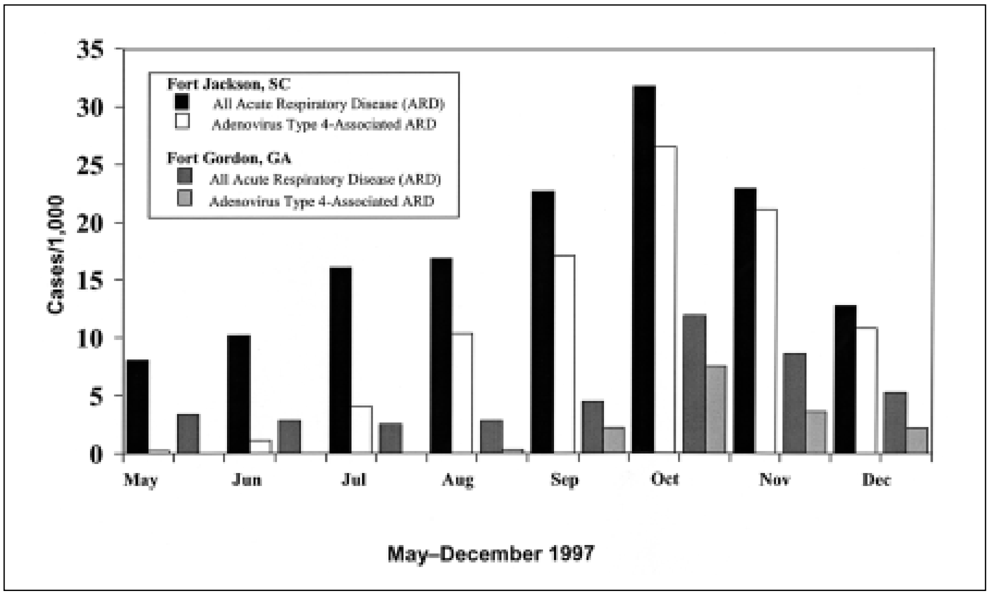

Figure 1. Numbers of introduced and secondary cases of adenovirus type 4-associated acute respiratory disease (ARD) at Fort Gordon, Georgia, USA, in soldiers who initially trained at Fort Jackson, South Carolina, August through December 1997.

causative agents were isolated from the 62 persons who were adenovirus type-4 negative. This resulted in an identifiable cause of ARD in 90 (75.6%) soldiers. Twenty-seven of the other agents were viruses. These included eight isolates of herpes simplex; eight of adenovirus type 21; four of parainfluenza type 3; five of adenovirus type 2; one of influenza B; and one mixed infection of adenovirus types 2 and 3. Six other trainees were positive for *Streptococcus pyogenes*, Group A. Two trainees with mixed infections of *S. pyogenes*, Group A, and adenovirus type 2 and adenovirus type 4, respectively, were identified.

The patient with the initial adenovirus type 4 isolate on August 19, 1997, had arrived from Fort Jackson on June 20, 1997. Twenty-five (34.2%) of the 73 cases of adenovirus type 4-associated ARD were classified as introduced; 48 (65.8%) were classifed as secondary (Figure 2). Of the introduced cases, 22 (88.0%) were in trainees from Fort Jackson. The remaining three were in trainees from three basic training sites, who arrived at Fort Gordon on October 15 or later. Twelve of the trainees from Fort Jackson who had introduced cases had onset of symptoms 1 day before arriving at Fort Gordon (day –1) to 4 days after arrival (day+4). The earliest identified, introduced cases were in trainees who arrived at Fort Gordon on August 29. One became ill the day of arrival, and the second, 3 days after arrival.

The outbreak persisted until December 12, 1997, when the last adenovirus type 4 isolate was identified as a secondarily acquired infection in an advanced training soldier who had not trained at Fort Jackson (Figures 1, 2).

### Conclusions

Adenovirus type 4 was not isolated in 180 non-ARD patients cultured during April and May, indicating that the virus was not then circulating widely, if at all, at Fort Gordon. We stopped culturing non-ARD patients because of the increased workload in the medical center's virus laboratory caused by the ARD outbreak at Fort Jackson. Adenovirus type 4 was not isolated from ARD patients at Fort Gordon until August 19. The index patient had reported to Fort Gordon 58 days before onset of illness, a period much longer than the usual incubation period for adenovirus type 4; we classified that case as secondary, although it is possible that the patient was infected at Fort Jackson and excreted adenovirus type 4 at Fort Gordon without symptoms of clinical illness until August. Virus watch programs recognize infections with little or no illness and brief excretion of virus as well as persistent infections often characterized by illness and periods of intermittent virus excretion that can exceed 100 days (7,8). We were unable to identify the trainee who introduced adenovirus type 4 to Fort Gordon from a basic training center.

Our data are consistent with the introduction of adenovirus type 4 (with subsequent virus circulation) to Fort Gordon during June to

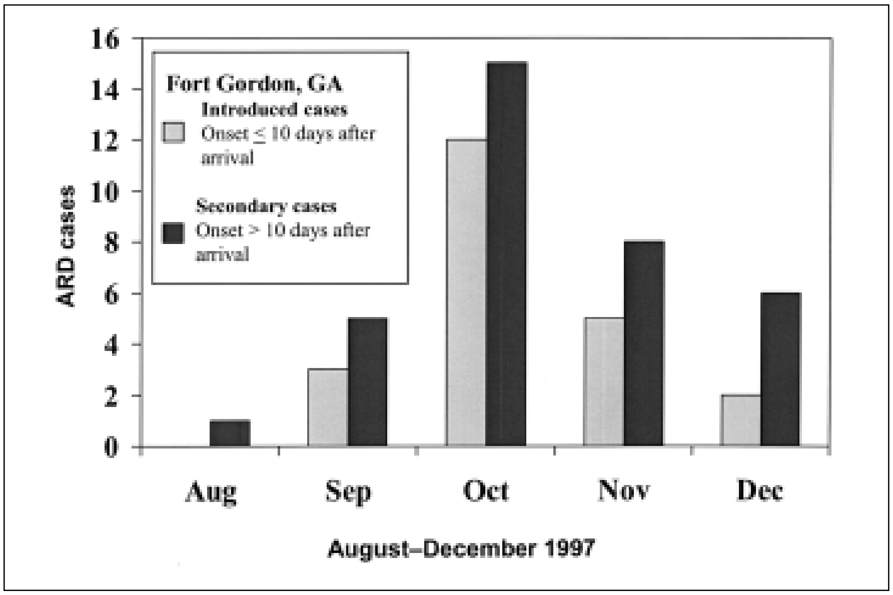

Figure 2. All acute respiratory disease (ARD) and adenovirus type 4-associated ARD incidence rates (cases/ 1,000 trainees/month) at Fort Jackson, South Carolina, and Fort Gordon, Georgia, May through December 1997.

August, probably by a trainee from Fort Jackson. The likely spread of adenovirus type 4 from Fort Jackson to Fort Gordon is supported by the temporal relationship between the outbreaks at the two sites and the laboratory-proven adenovirus type 4 infections in soldiers arriving at Fort Gordon from Fort Jackson. Immunizations for adenovirus type 4 ceased at Fort Jackson and elsewhere in the army on April 1, 1997, and the adenovirus type 4 ARD outbreak in Fort Jackson basic trainees began in May (1). Since basic training was 8 weeks, the first cohort of nonimmunized soldiers arrived at Fort Gordon at the beginning of June. Strain variations have been described in isolates of adenovirus type 4 (9). Molecular identification procedures were not available in military laboratories at the time of this outbreak, and the cost of molecular characterization or other tests to compare isolates from basic training centers and Fort Gordon were beyond the study budget.

The adenovirus type 4 outbreak in the advanced training population at Fort Gordon differed from the much larger outbreak in Fort Jackson basic trainees. At Fort Jackson, adenovirus type 4 accounted for more than 90% of ARD hospitalizations toward the end of the 1997 epidemic (3). At Fort Gordon, isolation of adenovirus type 4 occurred less often: <50% of soldiers hospitalized with ARD at Fort Gordon had an adenovirus isolated. Among the remaining 50%, a potential respiratory pathogen was identified for 53.2%. Laboratory tests to identify *Mycoplasma pneumoniae* and *Chlamydia pneumoniae* as causes of ARD were not available for this study (10).

ARD rates were much lower at Fort Gordon than at Fort Jackson (Figure 2). At its peak, the Fort Jackson outbreak produced an attack rate of 26.45 cases per 1,000 soldiers per month. The highest attack rate at Fort Gordon in advanced training soldiers was 7.50 cases per 1,000 soldiers per month. Various factors including naturally acquired immunity from exposure to outbreaks during basic training, less crowded housing in advanced training (two-person rooms in advanced training versus open barracks in basic training), and less physical stress in the highly technical Fort Gordon training program may have limited the magnitude of that outbreak.

The outbreak among advanced training soldiers at Fort Gordon supports the hypothesis that the spread of adenovirus type 4 from one training site to another can occur. This outbreak also demonstrates that adenovirus type 4-associated ARD occurs in advanced training soldiers and results in hospitalizations. Since a new adenovirus vaccine manufacturer has not yet been identified, the U.S. military may not have adenovirus type 4 or 7 vaccines until 2004 or beyond. Surveillance must be conducted at basic and advanced training centers with special consideration given to studying adenovirus transmission. Understanding the variables associated with virus spread could lead to effective nonvaccine interventions.

### Acknowledgment

The authors thank Johnnie Conolly of Moncrief Army Community Hospital, Fort Jackson, South Carolina, for her invaluable and expert technical assistance.

Dr. McNeill is a district health director with the South Carolina Department of Health and Environmental Control. He was formerly Chief, Preventive Medicine Service, Dwight David Eisenhower Army Medical Center, Fort Gordon, Ga. His special interest is the laboratory-based surveillance of communicable diseases.

### References

1. McNeill KM, Hendrix RM, Lindner JL, Benton FR, Monteith SC, Tuchscherer MA, et al. Reemergence of adenovirus type 4-associated acute respiratory disease in U.S. army trainees: report of a large, persistent epidemic. Emerg Infect Dis 1999;5;798-801.
2. ARD Surveillance Update. Medical Surveillance Monthly Report, Army Medical Surveillance Activity 1998;4:13.
3. Lennette DA. General principles for laboratory diagnosis of viral, rickettsial, and chlamydial infections. In: Lennette EH, Lennette DA, Lennette ET, editors. Diagnostic procedures for viral, rickettsial, and chlamydial infections. 7th ed. Washington: American Public Health Association; 1995. p. 3-25.
4. Reed LJ, Muench H. A simple method of estimating fifty percent endpoints. American Journal of Hygiene 1938;27:493-7.
5. Foy HM. Adenoviruses. In: Evans AS, Kaslow RA, editors. Viral infections in humans. 4th ed. New York: Plenum Press; 1997; p. 119-38.
6. Gaydos CA, Gaydos JC. Adenovirus vaccines, 1999. In: Plotkin SA, Orenstein WA, editors. Vaccines. 3rd ed. Philadelphia: WB Saunders; 1999. p. 609-28.
7. Fox JP, Brandt CD, Wassermann FE, Hall CE, Spigland I, Kagan A, et al. The virus watch program: a continuing surveillance of viral infections in metropolitan New York families. VI. Observations of adenovirus infections: virus excretion patterns, antibody response, efficiency of surveillance, patterns of infection, and relation to illness. Am J Epidemiol 1969;89:25-50.

8. Fox JP, Hall CE, Cooney MK. The Seattle virus watch. VII. Observations of adenovirus infections. Am J Epidemiol 1977;105:362-86.
9. Crawford-Mikoza LK, Nang RN, Schnurr DP. Strain variation in adenovirus serotypes 7 and 7a causing acute respiratory disease. J Clin Microbiol 1999;37:1107-12.
10. Gray GC, Callahan JD, Hawksworth AW, Fisher CA, Gaydos JC. Respiratory diseases among U.S. military personnel: countering emerging threats. Emerg Infect Dis 1999;5:379-87.
